# Dental Materia Medica

**Published:** 1865-12

**Authors:** Geo. Watt


THE
DENTAL REGISTER OF THE WEST.

VOL. XIX.] DECEMBER, 1865. [NO. 12.

### Original Essays and Communications.

### DENTAL MATERIA MEDICA.

BY GEO. WATT.

AT the late meeting of the AMERICAN MEDICAL ASSOCIATION, the subject of specialties and specialists in medicine received a good degree of attention, the reports on the subject, and the discussions that followed, going to show a decided want of harmony in the views of the members. Two members of the committee on medical specialties presented each an individual report, in which decidedly different opinions are set forth; and another member of the committee is represented as disagreeing with both, if my memory is correct.

An earnest discussion followed the reports, which were finally referred to the committee on ethics.

Dr. H. R. Storer, one of the committee, in his report takes the position that specialists have already vindicated their position, and are the leaders of the profession. This sentiment seems to have met with decided opposition. Indeed, from any notice of the proceedings that I have seen, I infer that specialties were not regarded with favor. A motion was made to establish a section on opthalmic medicine, which was rejected, after considerable discussion.

The very able lecture of Professor Brainerd, delivered to the American Dental Association, at the late meeting in Chicago, if as widely circulated through the medical, as it has been through the dental profession, would probably do much to change the tide of feeling. As it has been extensively published in the dental periodicals, it would be well for dentists to call the attention of physicians to it, "as unto a light that shineth in a dark place, till the day dawn and the day-star (of professional progress) arise in their hearts." For, I regard it as evident, that the position of Dr. Storer is, to a considerable extent true even now; and that it will be true, ere long, in its widest sense, is just as evident, unless the specialists prove false to their callings and positions. The farmer of a thousand acres is not expected to cultivate as carefully, or with as much success, in proportion to the space, as the market gardener; and the horticulturist is presumed to know more of flowers than the agriculturist. But if the gardener is ignorant of the properties of soils and manures, and of the plainer principles of vegetable physiology, and, especially if he is indolent and negligent, he will, undoubtedly, be surpassed by the intelligent, industrious farmer, even on flowers.

But that specialists may be "leaders of the profession," as claimed for them by Dr. Storer, several things are necessary. No man can be fit to practice on living organs, without a knowledge of the anatomical structure of these organs, and of their physiological and pathological conditions; as well as of their relations to other organs, and to the system at large. This means, in short, that the specialist should have an intimate acquaintance with all the departments of medical science. "Only this, and nothing more;" and, certainly nothing less.

Our dental colleges recognize this fact, at least to a good degree, in their *curriculi.* These, however, will have to be farther extended, and rendered more minute, as the profession progresses toward perfection. The notion is yet far too prevalent, in our profession, that some departments of medical science are of but little importance to the dentist. This idea has been carried from the people, who entertain the opinion that "anybody can pull a tooth," and believe that it requires much less talent and skill to be a dentist than a physician. A case that occurred in my practice will illustrate this: A lady requested me to extract a tooth which was but slightly decayed. The pain was paroxysmal, and very severe. After a short, but careful examination of the case, I wrote her a prescription, and appointed a time to fill the tooth. She followed the prescription, and obtained early and complete relief, and returned according to appointment. After the tooth had been filled, we began a friendly conversation. We had been friends from childhood; and, hence, she had a thorough acquaintance with my professional history. The ability to prescribe successfully for her case, she imputed, not to dental, but to medical knowledge. She thought it was very sad, that, after all my toil and study to acquire a knowledge of medicine, I had lost all its advantages, and was obliged to throw it away. To a reply that it had not been thrown away, she said, "But it is of no use to you now" I insisted that I had still constant use for it. "Well, only for part of it," she replied. "No, for all of it," said I. "Oh, hush!" said she, "you needn't pretend you have use for the old woman department, any way." "But for my knowledge of that," said I, "I might have extracted your tooth." Her reply was a blush, and the statement, "If my sons become dentists, they *must* be physicians, too." Thus was this lady enlightened; and her appreciation of the wants and capabilities of the dental profession has kept alive ever since. But her first views are a fair representation of those entertained by the public.

While it is true that a knowledge of the whole field of medicine should be possessed by the specialist, still, in the present state of our specialty, it may be well enough to write and speak with reference to partial attainments. For even in that perfected state of our profession, to be found in the "good time coming," monongraphs, and other works on special subjects, will be wanted — will be even more needed than now; for, by that time, if not before, our specialty will probably be divided into two or more specialties, or *sub-specialties*, if you prefer the term.

Something in the shape of a text-book on dental anatomy, dental physiology, dental pathology, dental materia medica and therapeutics, is a pressing want of our profession, a want that is felt by every live member in it. Now, I have not the slightest intention of writing a text-book on any of these subjects, nor, indeed, on any other; but, till something of the kind is written, a few articles on the medicines principally used by dentists, may not be amiss; and these I propose to write, if there be no farther failure of nerve and muscle.

A dental classification of the medicines used in our profession, if practicable, is of doubtful propriety. In the main, I prefer to notice them individually.

It is an incontrovertible fact that, by a majority of the profession, most, if not all of these medicines are used empirically. But little is known of their composition, preparation, or *modus operandi*; and, usually, as little of their adulterations, tests, and modes of purification. Somebody ought to give light in this direction; — it should have been given long ago. I have waited, and watched, and "wondered that there was no man" to do it. I wait and watch no longer; and will leave others to wonder at my presumption in trying to teach that of which I know so little. My hope is that, in trying to furnish thoughts for others, I may gain some new and useful ideas myself.

There is a want of clearness in our profession, as to the line of demarkation between the specialist and the general practitioner. In the management of diseases of the mouth, constitutional treatment is often necessary. Now, it is the opinion of some that the patient, under such circumstances, should be referred to the family physician for the constitutional treatment, while that which is purely local comes within the province of the dentist. The mere mention of this question demonstrates the necessity of a general knowledge of medical science, on the part of the specialist.

What is to be done if the family physician be a quack? How soon will a patient, under his treatment, be prepared for an operation? It is strange that any should entertain the idea that the whole treatment does not come within the duties of the dentist. But if the dentist is not competent to conduct the general treatment, the family physician, or *some* physician must be consulted; and, in many cases, even the best educated dentist will do well to consult a good physician. Physicians consult with each other, with advantage to themselves and their patients. A dentist is no less likely to need advice than a physician.

If the few articles which I hope to be able to write, and to which this is intended as a crude introductory, shall prove useful to the younger members of our profession, I shall be content. It is not expected that they will be instructive to all. Their shortcomings may stimulate some abler pen; and thus, the profession may be doubly benefitted.

